# Barriers to practicing General Practice in rural areas – Results of a qualitative pre-post-survey about medical students during their final clinical year

**DOI:** 10.3205/zma001196

**Published:** 2018-11-15

**Authors:** Kathrin Ludwig, Corina Machnitzke, Thomas Kühlein, Marco Roos

**Affiliations:** 1 Friedrich-Alexander-Universität Erlangen-Nürnberg (FAU), Institut für Allgemeinmedizin, Erlangen, Germany

**Keywords:** general practice, practical year, barriers to setting up practice, qualitative research

## Abstract

**Objective: **At the end of the Practical Year (PY), medical students decide on a specialization. Individual motivational factors and barriers play a central role in the choice of the subsequent subject area and the place of establishment (city/country). The aim of this study was to document the barriers of PY students within the General Practice (GP) tertiary elective over time.

**Methodology: **Two guided interviews were conducted with each participant (N=19) as part of qualitative process monitoring – a pre-interview at the start and a post-interview after completion of the PY. Evaluation of the interviews was based on Grounded Theory.

**Results: **13 barriers could be deduced from the 38 interviews. The most frequently cited barriers were “expected workload”, “recreational opportunities”, “work-life balance” and “compatibility with family”. 13 of the participants were firmly committed to continuing GP training, 12 of whom aspired to opening a practice in a rural area. Another three were considering GP training, three had decided against it after the PY. After the PY, some of the previously anticipated individual barriers were now perceived in a more differentiated manner as a result of practical experience. The barriers “work-life-balance”, “compatibility with family”, “recreational opportunities” and “infrastructure” had been largely eliminated.

**Conclusion:** The PY General Practice Tertiary itself appears to have a positive impact on the individual barriers expressed before the PY. Targeted experience with a PY in General Practice at the end of study seems to be a solution to increase the attractiveness of the subject.

## Zusammenfassung

**Zielsetzung:** Am Ende des Praktischen Jahres (PJ) entscheiden sich Medizinstudierende für eine Fachrichtung. Bei der Wahl des späteren Fachgebietes und des Niederlassungsortes (Stadt/Land) spielen individuelle Motivations- und Barrierefaktoren eine zentrale Rolle. Ziel dieser Studie war die Erfassung der Barrieren von PJ-Studierenden mit Wahltertial Allgemeinmedizin (AM) im zeitlichen Verlauf.

**Methodik: **Mit jedem Teilnehmer (N=19) wurden im Rahmen einer qualitativen Prozessbegleitung zwei leitfadengestützte Interviews durchgeführt – ein Prä-Interview zu Beginn, ein Post-Interview nach Abschluss des PJ. Die Auswertung der Interviews erfolgte angelehnt an die Grounded Theory.

**Ergebnisse:** Aus den 38 Interviews konnten 13 Barrieren abgeleitet werden. Die am häufigsten genannten Barrieren waren „erwartete Arbeitsbelastung“, „Freizeitmöglichkeiten“, „Work-Life-Balance“ und „Vereinbarkeit mit Familie“. Fest entschlossen zur Weiterbildung AM waren 13 der Teilnehmenden, wovon 12 eine ländliche Niederlassung anstrebten. Weitere drei zogen die Weiterbildung in Betracht, drei entschieden sich nach dem PJ dagegen. Nach dem PJ wurden einige der zuvor erwarteten individuellen Barrieren durch die Praxiserfahrung nun differenzierter wahrgenommen. Die Barrieren „Work-Life-Balance“, „Vereinbarkeit mit Familie“, „Freizeitmöglichkeiten“ und „Infrastruktur“ wurden weitestgehend abgebaut.

**Schlussfolgerung:** Das PJ-Tertial AM selbst scheint einen positiven Einfluss auf die vor dem PJ geäußerten individuellen Barrieren zu haben. Gezielte Erfahrungen mit einem PJ in der AM am Ende des Studiums zu ermöglichen scheint ein Lösungsansatz zur Steigerung der Attraktivität des Faches zu sein.

## 1. Background

The nationwide provision of GP care in rural areas presents a challenge in the German health care system [1]. During their education, medical students are often exposed to pejorative views of General Practice (GP) in other specialist disciplines [2], [3]. A positive perception of a subject, arising from different determinants that can be modeled, seems to be a predictor for the subsequent choice of this discipline [4]. 

Even young physicians, who have already decided in favor of General Practice, are aware of barriers to setting up practice in rural areas. The aspects most frequently discussed in the literature are unfavorable conditions for reconciling work and family life. These include poor job prospects for the life partner, poor infrastructure, the financial risks involved in setting up practice, suspected low earning potential and an expectation of a higher workload and social responsibility [5], [6], [7], [8], [9]. In addition, students seem to have a distorted perception of rural practice [10]. This leads to the assumption that some of the barriers of students to undertaking General Practice in rural areas can be explained on the one hand by a lack of practical experience and on the other hand by distorted perception. 

Through various measures, politicians and committees of medical self-government are trying to create incentives for practicing medicine in rural areas [11], [12]. In addition, the Master Plan for Medical Studies 2020 adopted measures to modernize medical studies. Part of this modernization is improved integration of General Practice in the medical curriculum. In addition, the introduction of a country doctor quota at the provincial level and a mandatory quarter of outpatient SHI medical care during the Practical Year (PY) should counteract the shortage of young doctors in rural areas [11]. 

The qualitative monitoring of PY students in the GP Tertiary Elective presented here in rural GP surgeries intended to document changes in the attitudes towards the subject area and towards potential future practice in rural areas over time. 

## 2. Methods

To explore individual expectations and experiences, a qualitative approach was chosen. The participants (TN) were interviewed in two guideline-based one-to-one interviews. The interviews were conducted at the beginning of the Practical Year (Pre) and at the end (Post).

### 2.1 Selection of PY students 

In the period from October 2014 to July 2017, PY students taking the GP elective were invited to participate in the study at the medical faculties in Erlangen, Würzburg and Regensburg (n=37). All PY students from Erlangen (n=33) were also offered a place in a rural teaching practice in combination with an expense bonus of €650 per month (in the GP tertiary). 

#### 2.2 Selection of rural teaching practices 

For the training of PY students in GP, the GP Institute of the University Hospital Erlangen has 33 teaching practices at their disposal. All medical student trainers must regularly take part in various CPD courses on medical education. For the study, eight practices in rural areas (two individual practices and six group practices) were selected. Inclusion criteria were the rural location (distance to the nearest hospital, density of additional outpatient specialist care), sufficient size and equipment, as well as positive evaluation results. The teachers of the eight rural practices completed a separate CPD course in medical eduction in December 2014. This included learning objectives for the PY GP Tertiary, tips for organizing and integrating the students into the daily routines of the practice, as well as practical feedback training. 

#### 2.3 Guideline Development

Based on the available literature, different guidelines were developed for the pre- and post-interviews. The pre-interviews were divided into the topics “Individual perception of General Practice”, “General Practice at university”, “Choosing the PY General Practice Tertiary” and “Choosing a teaching practice in a rural area”. The post-interviews focused on “Experiences in the General Practice elective", “Individual perception of General Practice” and “Future planning”. The key questions were supplemented by further questions, some of them spontaneous and partly based on the results of the pre-interview survey, in order to make it easier for the interviewers to adapt flexibly to the course of the interview and to increase the depth of the interviews. In the course of data collection, the guidelines were adapted to the evaluation results and thus developed further. 

#### 2.4 Data collection

The interviews lasted 33 minutes on average (13-68 minutes). They were conducted in person or over the phone by a medical doctoral student (KL) and a non-medical scientist (CM). The interviews were digitally recorded, transcribed and anonymized. 

#### 2.5 Data Analysis

The data was evaluated based on “Grounded Theory” [13]. The evaluation was carried out using the RQDA program. Data collection, coding and evaluation followed the “constant comparison” method [13]. In terms of intersubjective traceability [14], coding and evaluation were carried out in parallel and independently by three authors (KL, CM, MR). To begin with, small-step open inductive coding of the first interview with generation of first concepts and hypotheses was carried out. By encoding further interviews, a category system emerged through the process of axial and selective coding [13]. The interviews continued beyond saturation until all included PY students had been interviewed twice. In a consensus round, the codes were compared by the authors and a common category system was developed. 

## 3. Results

The results present the statements of the pre- and post-interviews of 19 PY students (4 male, 15 female, no dropout). Of these, 13 participants completed their PY in one of the selected rural practices, six participants in urban practices. After the PY, a total of 13 participants had definitely decided in favor of specialization in GP (with 12 participants to start training in a clinic, one in a rural practice). Three took specialization in GP into consideration and a further three decided against it. 

A rural place of work was conceivable for a total of 13 participants (PY rural practice: 8 participants, PY urban practice: 5 participants). For six participants this was not conceivable (PY rural practice: 5 participants, PY urban practice: 1 participant). Of these, three participants preferred setting up practice in an urban area (PY rural practice: 2 participants, PY urban practice: 1 participant) and another three participants preferred work in another specialization (all from PY rural practice). Only two participants who had completed the PY in a rural practice ultimately decided against setting up practice in the countryside. One participant for personal reasons and the other as they had already settled and started a family. A description of the sample is shown in Table 1 (Tab. 1). All participants recommended the GP tertiary elective regardless of the later subject selection for their fellow students. Only one limited the recommendation to students interested in GP. The influence of financial compensation on the choice of a rural practice was given by the participants as being between 0 and 50%. 

In the interviews, we asked the students about the barriers they perceived to General Practice in rural areas. There were 13 barriers, most commonly “anticipated workload”, “effects on the family”, “recreational opportunities” and “work-life balance”. The barriers could be assigned to two main categories in the evaluation. For three participants the barriers “External factors: finances, bureaucracy, health care system”, “diagnostic uncertainty”, “one-sidedness of the GP profession” and “proximity in patient contact” were decisive for turning their back on GP. In the following, after each quote the TN number, interview (pre, post), training inclination (WB) (Certain: WB+, Maybe: WB~, No: WB-) and the inclination towards setting up in a rural area (Rural+/Rural-) are indicated.

### 3.1 Barriers to choosing General Practice as a specialization

#### 3.1.1 “Expected Workload”

In the pre-interviews, participants suspected a higher workload compared to other subject areas. Following practical experience, this was confirmed, but was more differentiated due to more flexible working hours. 

“Well, of course it’s a lot of time you end up investing, [...] But in return you also have the advantage of being free at lunch time, for example.” (TN 2 Post, WB+, Rural+)

##### 3.1.2 “External Factors: finance, bureaucracy, health care system”

While in the pre-interviews some of the participants were expecting inadequate and sometimes even poor pay for GP activities, no more comments were made on the income of a GP in the post-interviews. Instead many statements were made on the required business knowledge, the bureaucratic burden of a practice and restrictions on medical work by the health care system.

“Well, basically you end up running a medium-sized company, but never having learned anything of business admin [...], never anything about accounting, that’s shocking.” (TN 5, Post, WB-, Rural-)

##### 3.1.3 “Diagnostic Difficulty & Uncertainty” and “One-Sidedness of the GP Occupation”

The work of a rural GP and, in the eyes of one participant, limited range of activities led to two participants turning their backs on the option. 

 “And then you leave it to wait-and-see and then next time around it’s gone and you never know what it was.” TN 5 Post, WB-, Rural-)

For the majority of the participants, however, these two factors were a motivation for a career in General Practice. 

“[...] and using guidelines and evidence-based structures, you were able to gain confidence in dealing with patients. [...] you’re a doctor on your own and you can be a very good doctor.” (TN 8, Post, WB+, Rural+)

#### 3.2 Barriers to General Practice in rural areas

##### 3.2.1 “Expected Workload”

In the pre-interviews, rural practice was seen as more intensive and more time-consuming than other fields. 

“[...] this huge amount of time each week, that can really finish you.” (TN 13, Pre, WB+, Rural+)

In the post-interviews, this was relativized by the practical experience, especially in the rural practices. The workload fluctuated depending on the organization of practice structures. Chaotic daily schedules stood in contrast to well-functioning teamwork or practice organization. 

##### 3.2.2 “Leisure opportunities”

One very common suspicion voiced in the pre-interviews regarding the causes of the lack of young rural doctors was the assumption that cities are probably more attractive due to a more varied offer of recreational opportunities. 

“Maybe in the countryside, to put it bluntly, there’s too little going on.” (TN 15, Pre, WB+, Rural+)

In the post-interviews, most participants were showing a willingness to compromise on the distance between home and work, as well as the accessibility of urban activities. Travel times up to 45 minutes were considered acceptable. 

##### 3.2.3 “Work-Life Balance”

In the pre-interviews, many of the participants associated working in a rural area with an unsatisfactory work-life balance due to patients being able to contact you around the clock. This was not confirmed by the participants in the post-interviews. It turned out that especially group practices were perceived to offer a good work-life balance. The separation of home and workplace was a good solution to this problem. 

“Therefore, I think that in the context of group practices, [...] that it is definitely possible to have a pleasant work-life balance as a GP.” (TN 17, Post, WB+, Rural+)

##### 3.2.4 “Compatibility with family”

Compatibility with having a family was an important influencing factor for the participants. In the pre-interviews there was the expectation that the work in a practice would be more compatible with having a family than working in a clinic. This was to be put to the test in the PY and was subsequently confirmed, based on the possibilities of group practice, part-time work and more flexible working hours. 

“I think, especially as a female GP, [...] it’s easier to manage having a family than, for example, as a senior physician in a clinic, with loads of weekend shifts, emergency shifts, being on-call etc.” (TN 12, Post, WB-, Rural-)

Nevertheless, one criticism that was mentioned in the post-interviews was that settling in the countryside could also fail due to having to move a whole family.

“And that’s usually the reason why it fails, ’cos the family has to tag along. [...] especially your partner, but also the kids [...] they got their school, their friends, making it less likely you’ll move.” (TN 1, Post, WB+, Rural+)

##### 3.2.5 “Partner’s Job”

A partner requiring job opportunities is another inhibiting factor when considering a career in a rural practice. 

“That’s what we always said, if the partner can’t come, you won’t go.” (TN 1, Post, WB+, Rural+)

##### 3.2.6 “Patient structure”

With regards to the patient structure, predominantly elderly patients were expected in the pre-interviews. After the tertiary elective, however, the participants reported a broad distribution of the patient population. 

##### 3.2.7 “Infrastructure”

The infrastructure of rural areas was also seen as a limitation in the pre-interviews. On the one hand, few supermarkets, secondary schools leading to university and kindergartens, and on the other hand by poor public transport.

*“The kids may have to travel far to school or to any sports or music lesson.” (TN 8, Pre, WB+, Rural+)*

Having experienced work in rural practices, this barrier was considered hardy worth mentioning in the in the post-interview. 

##### 3.2.8 “Proximity in patient contact” and “Integrating into a rural community”

A particularly close doctor-patient relationship in rural practices was already expected in the pre-interviews. However, only a small section of the participants later saw this as a barrier. 

“[...] such close, long-term patient contact [...] I’m not that kind of person.” (TN 5, Post, WB-, Rural-)

The medical profession was also seen as a way of integration into the community. 

“Well, I think, with a job that [...] really brings personal benefits for the locals [...] I think it’s easier.” (TN 5, Post, WB-, Rural-)

#####  3.2.9 “Large catchment area”

In rural practices, pre-interviews expected there to be a very large catchment area due to the low density of doctors and long trips to carry out home visits and patients having to travel far to see a specialists or get to the nearest hospital. No uniform perception could be derived from the post-interviews. For one participant long distances were generally unattractive, another was accustomed to these due to their own rural origins. Another participant thought that although the distances were longer, the speed you could travel at was higher compared to similar travel distances in a large city.

A list of the barriers with further anchor citations is shown in Tab. 2 at the attachment 1 . 

#### 3.3 Difference of PY rural practice vs. PY urban practice

The “work-life balance”, “patient structure” and “integration in local community” barriers changed equally in both groups as described above. The barriers “workload”, “recreational opportunities” and “large catchment area” remained constant in the urban group, while they largely decreased in the PY rural practice group, partly due to compromise. 

The barriers “infrastructure” and “compatibility with family” were only mentioned in the post-interviews by the PY students of the rural group and had reduced as a result of their experiences in the PY. Also, “job of the partner” is only an important factor for the rural practice group and persists after the PY. 

The barriers “External Factors: finances, bureaucracy, health care system”, “diagnostic uncertainty”, “one-sidedness of the GP profession” and “proximity in patient contact” only played a role for the three participants, who ultimately decided against specializing in GP.

## 4. Discussion

### 4.1 Summary

Before the start of the PY GP Tertiary, clear barriers were perceived both to GP and setting up a practice in a rural area. After the PY, it became clear that the previously expected individual barriers were now perceived in a more differentiated manner and were partially completely eliminated by the practical experience. A comparison of the PY experience with personal inclinations led to a reassessment of the previously expressed barriers, often through reaching compromises. Especially the barriers “poor work-life balance”, “poor compatibility with family”, “lack of recreational opportunities” and “poor infrastructure” were largely reduced by the experience. The fact that all but three participants are interested in specializing in GP also suggests that the barriers to the subject can be minimized solely by experience in the PY and the resulting adjustment of perception. When examining the practice setup trend, it is surprising to note that most participants, both from rural and urban practices, prefer setting up in a rural area and only a few decide on setting up practice in the city for personal reasons.

#### 4.2 Comparison with the literature

The workload was perceived by our participants as high, but was accepted due to the flexible distribution of working hours during the week and the lack of weekend shifts. In addition, a well-regulated work-life balance was also perceived in rural practices. Similar findings were found in a study by Maenner et al. [8]]. He also mentions the high level of responsibility that an individual practice entails and describes cooperative practice models as an “alternative” [8]. In a survey among medical students of the Hartmannbund regarding practice setup, cooperative practice models were also preferred to setting up single practices [15]. After “financial risks”, compatibility with family life was one of the main reasons against setting up single practices. Family circumstances, including reconciliation of work and family life, are a key desirable of future GPs in relation to setting up practices in rural areas. Two surveys of young physicians on this topic confirmed that family circumstances were an important influencing factor, irrespective of the specialization [6], [7]. 

For many of our participants, the close doctor-patient relationship and chance to support whole families is one of the main motivators in choosing a specialist area. Patient-centered work has already been identified as the most important factor for future GPs [16]. Similar results were found in a study by Roos et al., in which young GPs saw the advantages of GP especially in the area of personal patient contact and a steady work-life balance [17].

The attractiveness of urban vs rural areas in our study was directly related to the personal willingness to drive to the nearest city to engage in a certain activity. With regard to the longer distances in rural areas, travel times of 30 minutes for home visits and the commute to work, and 10 minutes for travel to shops and educational facilities for children are also considered acceptable in the literature [9]. According to Niehus et al. “personal preferences” are also an important factor when deciding to set up practice in a rural area [5]. We were unable to deduce a connection between place of origin or socialization and the preference for setting up practice as other studies showed [5], [7]. 

Three participants decided to turn their back on specialization in GP as a result of their experience in the PY GP Tertiary. The reasons were the lack of business knowledge, the expectation of a low income and a high administrative effort and coincide with known barriers from the literature [5], [6]. An increase of bureaucracy in the medical workplace is also discussed in the literature [3] and does not seem to be an issue facing general practice in rural areas specifically. The desire for including business studies into the degree course was found amongst students by Niehus et.al. [5]. According to Steinhäuser et al. such knowledge reduces the fear factor from establishing a practice [9]. 

The life-partner’s career situation also represents a barrier to setting up practice in rural areas which is discussed in the literature [7], [9], [15]. More than 90% of mayors of urban and rural regions in Baden-Württemberg consider ensuring GP care to be a “task for local administration” [18]. The greatest potential lies in the promotion of “childcare, building land and support of the life partner in their job search” [18]. There is an “information deficit” amongst young physicians in training regarding the reality of life in such communities. Infrastructure has long been much better than expected by them [18]. Amongst our participants, this barrier also was reduced by work experience in a rural area. 

Practical experience in the GP Tertiary also seems to be useful as a baseline for future education in other subject areas. The flexible continuing education regulations allow young physicians to leave ultimate specialization goal open, as, for example, training in GP and internal medicine show a large overlap with 18 months of inpatient basic training in internal medicine [19]. In a recent qualitative work, Barth et. al. also showed that often use is made of the overlap of the two subject areas as a decision-making period [20]. 

#### 4.3 Strengths and Weaknesses

The present study refers to a small, regional cohort in northern Bavaria and therefore can only be generalized to a limited extent. However, it can serve as a guide for further multi-center studies. Recruitment took place only among the students registered for a GP PY, which could result in a selection bias. Recruiting among all PY students would only have been constructive for capturing cross-sectional barriers, not their changes during the GP Tertiary. The financial expense allowance for participants choosing rural practices may also have influenced the choice of practices. But this made the practicalities of transport or even changing residence more feasible for students. Despite the small sample size we see the design of a qualitative process monitoring by means of pre- and post- interviews as a strength. By flexibly adapting the questions to the flow of the conversation, a detailed exploration of their own expectations and barriers was achieved. 

## 5. Conclusions

Targeted job experience at the end of studies seems to break down barriers to GP in rural areas. Enable such targeted job experience could be an approach to increasing the attractiveness of GP in rural areas, thus providing an early incentive to young medical professionals to ensure future GP health care in rural areas. The framework conditions for the implementation of such work experiences are created by the catalog of measures in the Master Plan for Medical Studies 2020. With the support of politicians and municipalities, a wider implementation of such an approach would seem practicable and evaluable.

## Notes

The present study was part of a medical doctoral dissertation at the Friedrich-Alexander University, Erlangen-Nürnberg (FAU). 

## Funding

The study was funded by the Bavarian Ministry of Health and Care (G31a-G8060-2014/167-3). 

## Ethics Vote

The Ethics Committee of the Friedrich-Alexander University Erlangen-Nürnberg had no concerns regarding the study (Ref: 336_14 Bc).

## Competing interests

The authors declare that they have no competing interests. 

## Supplementary Material

Table 2: Classification system of the individual barrier factors (N=13) with anchor quotes from the interviews. After the quotes, the participant (TN), the interview (pre/post), the training inclination (WB) (Certain: WB+, Maybe: WB~, No: WB -) and the preferred region for setting up a practice (Rural+ /Rural-).

## Figures and Tables

**Table 1:**
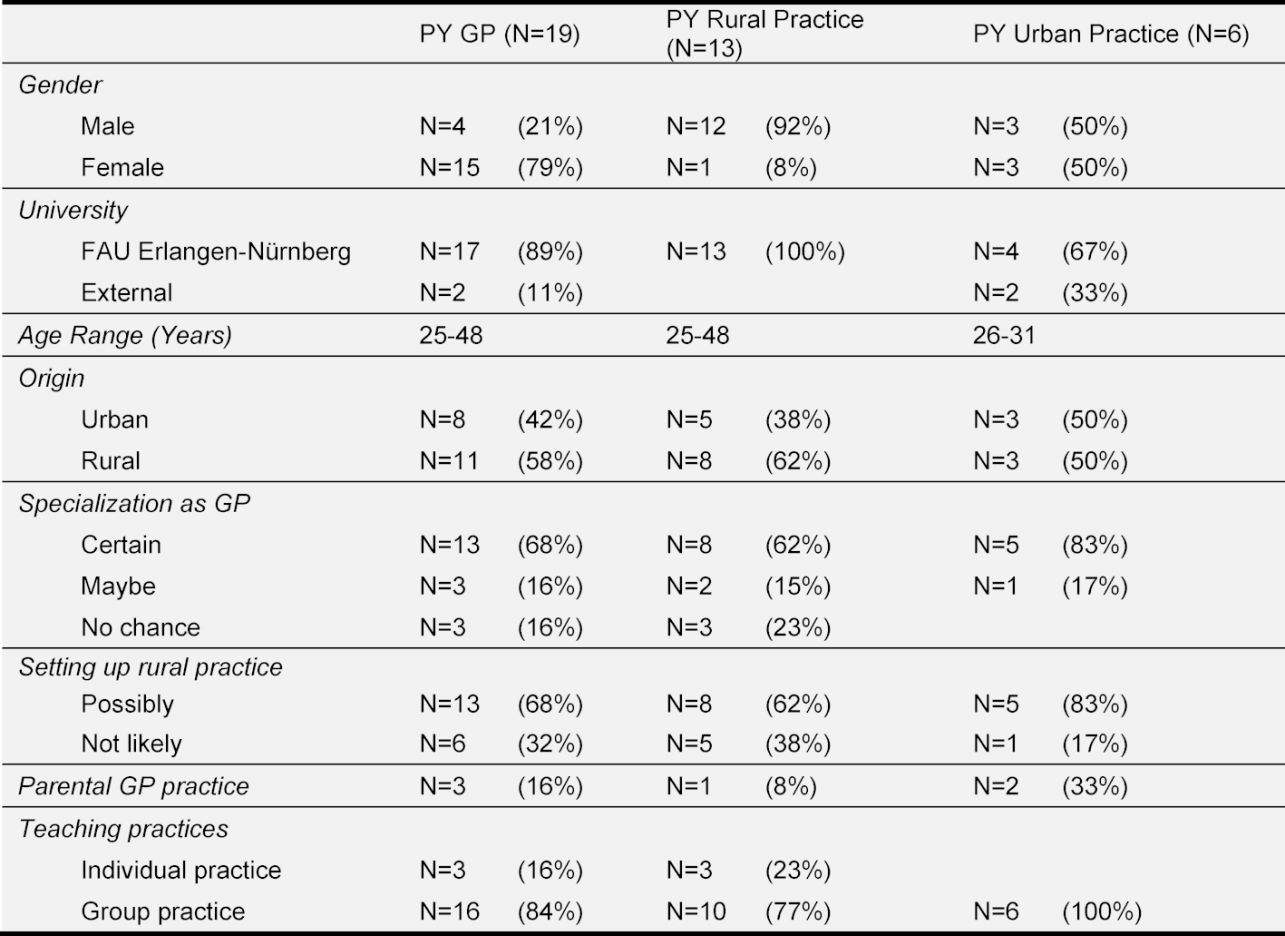
Sociodemographics, training tendency and teaching practices of the sample (N=19) divided into total sample PY GP, PY in rural practices (N=13), PY in urban practices (N=6)
