# Supplementary material for: Barriers to practicing General Practice in rural areas – Results of a qualitative pre-post-survey about medical students during their final clinical year
Source: GMS J Med Educ. 2018 Nov 15;35(4):Doc50. doi: 10.3205/zma001196 (PMC6278231; doi:10.3205/zma001196)
Supplement: Table 2: Classification system of the individual barrier factors (N=13) with anchor quotes from the interviews. After the quotes, the participant (TN), the interview (pre/post), the training inclination (WB) (Certain: WB+, Maybe: WB~, No: WB -) and the preferred region for setting up a practice (Rur [file JME-35-50-s-001.pdf]

Categories and Barriers	Anchor quotes from the interviews

Barriers to choosing General Practice as a specialization

Expected Workload

“I could imagine that many of them are simply discouraged, that there are fewer and fewer other doctors in rural areas and that the workload could possibly end up being even higher.” (TN 12, Pre, WB-, Rural-)

“I think you really work extremely hard. And if someone shows up at the practice, you can’t just say, ‘Naw, I’m done for the day, I’m going home now.’” (TN 16, Pre, WB+, Rural-)

“Well, of course you invest a lot of time, has to be said, and telling people otherwise would just be nonsense. [...] But on the other hand for instance at lunchtime you actually get time off. Well, I mean, it’s just a different way of life compared to many other professions.” (TN 2, Post, WB+, Rural+)

“Well, I found it rather exhausting, to be honest. [...] And the office hours were quite long, sometimes up to 7 or 8 in the evening. [...] But you got Wednesday afternoon off, which was very nice.” (TN 11, Post, WB-, Rural-)

External Factors: Finance, Bureaucracy, Health care system

“Your salary is definitely a factor. Yes, you don’t earn a fortune and having a career isn’t really an option either and that’s a factor for some folk” (TN 8, Pre, WB+, Rural+)

“What would discourage me a little bit of course would be the financial aspect.” (TN 17, Pre, WB+, Rural+)

“Well, basically you end up running a medium-sized company, but never having learned anything of business admin, never anything about accounting, that’s shocking.” (TN 5, Post, WB-, Rural-)

“And I also find the whole system [...] simply unfair that somehow a GP who gets the hang of the system better and uses codes in a smarter way but spends less time with the patients has more money in the end compared to someone who’ does it the other way round.” (TN 5, Post, WB, Rural-)

“I really have to point out that I was extremely put off by the whole bureaucracy.” TN 5, Post, WB-, Rural-)

“So there’s this level of stress, you know, and you really just work work and work. Um, yeah, that scared me off sure enough. The fact that one simply does not have enough time for the patients [...] through the budgeting of the health insurers means that in most practices it’s simply not possible to do much different .” (TN12, Post, WB-, Rural-)

One-sidedness of being a GP

“Well, I got a lot more respect for general practice. Especially because I think you have to cover the whole range, have very broad knowledge.” (TN 6, Post, WB+, Rural+)

“[...] how to gain certainty in some patient examinations. [...] Or how to become surer in dealing with patients using guidelines and evidence-based structures. [...] You’re a doctor on your own and you can be a very good doctor.” (TN 8, Post, WB+, Rural+)

“It really is a bit a case of same old, same old, that you end up seeing. [...] there is rarely something really new. [...] So somehow I felt better working in internal medicine.” (TN 11, Post, WB-, Rural-)

Diagnostic difficulty & uncertainty

“And then you leave it to wait-and-see and then next time around it's gone and you never know what it was.” [...] I always found a bit unsatisfactory.” (TN 5 ,Post, WB-, Rural-)

Barriers to GP provision in rural areas

Expected Workload

Categories and Barriers	Anchor quotes from the interviews
	"Of course, you have all sorts of duties and there's no new people in rural areas, so it just ends up being more and more and ever more." (TN 2, Pre, WB+, Rural+)<br><br>*"The negative, as I said, this immense amount of time you spend every week, which can really break you, I think." TN 13, Pre, WB+, Rural+)*<br><br>*"Well, if you spread the burden across many shoulders, then it was no problem at all to get everything done. [...] So, everyone's in the team, everyone has a specialism [...] So, that impressed me a lot, also the practice, the atmosphere, the way of working - that was just a great example. How it can work." (TN 1, Post, WB+, Rural+)*<br><br>*"But, if you run a group practice, I got the impression that it is quite doable [...] always changing, sometimes one, sometimes the other." (TN 2, Post, WB+, Rural+)*
Recreational Opportunities	
	"Maybe out in the countryside, to put it bluntly, there's too little going on. No idea. Too few recreational activities. Few discos, no parties, bars, whatever." (TN 17, Pre, WB+, Rural+)<br><br>*"For me, it would be attractive to work out in the country, but it would be important for me to be able to get into town within half an hour by car." (TN 19, Pre, WB+, Rural+)*<br><br>*"Half an hour's driving time would be okay, I think." TN 10, Post, WB~, Rural+)*<br><br>*"Compromise between city accessibility and rural life." (TN 8, Post, WB+, Rural+)*<br><br>*"I think here you just have a wider range of leisure activities. And that just is more my thing. I grew up out in the country, as soon as I was old enough I went to the secondary school in the city. At the time, I liked the city better than the countryside. I just feel a little better here." (TN11, Post, WB-, Land-)*
Work-Life Balance	
	"One thing I just can't imagine doing is um working 24/7, 365 days a year." (TN 1, Pre, WB+, Rural+)<br><br>*"I think that when you're out in the village, you're often approached by patients, which is a bit annoying." (TN 11, Pre, WB-, Rural-)*<br><br>*"And later on when I have my own practice, I can see it being a bit tricky, especially out in the countryside, that patients just don't accept when you're done for the day." (TN 12, Pre, WB-, Rural-)*<br><br>*"Well, even a doctor [...] should also be able to go shopping without constantly being asked medical questions. Well, it definitely don't have to be that way." (TN 1, Post, WB+, Rural+)*<br><br>*"[...] if, as a doctor, you also live in the village where the practice is, that's what I imagine being very stressful." (TN 11, Post, WB-, Rural-)*<br><br>*"Therefore, I think that within a group practice or pooling, that it's definitely possible to have a pleasant work-life balance as a GP." (TN 17, Post, WB+, Rural+)*
Compatibility with family	

Categories and Barriers	Anchor quotes from the interviews
	“Also group practice, so you can combine it in a family-friendly way. [...] Since we’re planning a family and I think - or hope - that it IS possible to combine it with being a GP after all.” (TN 8, Pre, WB+, Rural+)<br><br>*“Well I think, especially as a female GP, maybe with your own practice, that it’s easier to manage family alongside than, for example, as a senior physician in a clinic, with many weekend shifts, emergency shifts, being on-call etc.” (TN 17, Post, WB+, Rural+)*<br><br>*“I’ve just been to such a group practice, and that was totally reassuring [...] that it’s very possible, for example, to combine having a family with a GP practice.” (TN 2, Post, WB+, Land+)*<br><br>*“And that’s usually the reason why it fails, ’cos the family has to tag along. [...] especially your partner, but also the kids [...] they have their school, friends, making it less likely you’ll move.” (TN 1, Post, WB+, Rural+)*
Partner’s Job	*“That perhaps your partner might not find a job right there, can’t find work.” (TN 8, Pre, WB+, Rural+)*<br><br>*“That’s what we always said, if the partner can’t come, you won’t go.” (TN 1, Post, WB+, Rural+)*
Patient Structure	*“In contrast, in the countryside, like with my gran in the village, where there are, no idea, 90% over 60.” (TN 8, Pre, WB+, Rural+)*<br><br>*"That many aspiring physicians still think that GP care in rural areas means one thing: Geriatrics. Or you could call it geronto-psychiatric forest-and-meadow-medicine.” (TN 13 pre, WB+, Rural+)*<br><br>*“All sorts. That was very interesting, it really was everything from children up to wrinklies. Women, men, children, wrinklies, bright ones, idiots.” (TN 7, Post, WB~, Rural+)*
Infrastructure	*“Not three supermarkets nearby, not two secondary schools to choose from or three kindergartens.” (TN 2, Pre, WB+, Rural+)*<br><br>*“The kids may have to travel far to school or to any sports or music lesson.” (TN 8, Pre, WB+, Rural+)*<br><br>*“Yeah, well, of course, you can’t just go shopping, [...] but basic stuff, where, I believe, many are worried you have to jump in the car just because you forgot to get eggs [...] that’s not really a thing.” (TN 1, Post, WB+, Rural+)*<br><br>*“Because, maybe someday you can’t drive a car any more and then what? Then I’ll be stuck there ... yes, there’s a village shop, but that’s it.” (TN 4, Post, WB+, Rural-)*
Proximity of Patient Contact	*“I think if you don’t enjoy it and don’t like being close to your patients and your social environment then you can’t really be empathic either. I think it’s really a profession that requires a lot of passion.” (TN 17, Pre, WB+, Rural+)*<br><br>*“[...] such close, long-term patient contact, I just think I’m not that kind of person.” (TN 5, Post, WB-, Rural-)*
Integration into a Rural Community	*“Well, I think, with a job that [...] really brings personal benefits for the locals [...] I think it’s easier.” (TN 1, Post, WB-, Rural-)*<br><br>*“Well it’s definitely much harder for my friend. He did visit me there, but of course he’s not that much into it and he’s just worried... yes, that you’re just a bit alone, lost.” (TN 1, Post, WB+, Rural+)*

Categories and Barriers	Anchor quotes from the interviews
Large Catchment Area	
	“Specialists who are further away. More home visits, where you really have to drive to villages. Also probably not a lot of colleagues.” (TN 12, Pre, WB-, Rural-)<br><br>*“And I don’t want to have to travel for an hour to go to work every day, but [...] that’s actually the main reason why I don’t want to go to the country.” (TN 16, Post, WB+, Rural+)*
